# Solvent-Selective Complexity Reduction of Effluent Dissolved Organic Matter for ^1^H NMR Spectroscopy

**DOI:** 10.1021/acs.analchem.5c07560

**Published:** 2026-04-07

**Authors:** Sepehr Shakeri Yekta, Alex Enrich Prast, Mattias Hedenström, Tobias Sparrman, Luka Šafarič, Giacomo Carraro, Helena Rodrigues Oliveira, Thuane Mendes Anacleto, Annika Björn, Norbert Hertkorn

**Affiliations:** † Department of Thematic Studies - Environmental Change, 4566 Linköping University, 581 83 Linköping, Sweden; ‡ Biogas Solutions Research Center, Linköping University, 581 83 Linköping, Sweden; § Department of Chemistry, 8075 Umeå University, 901 87 Umeå, Sweden; ∥ 74351 Centro Federal de Educação Tecnológica Celso Suckow da Fonseca (CEFET/RJ), 20271 110 Rio de Janeiro, Brazil; ⊥ Unidade Multiusuário de Análises Ambientais, Instituto de Biologia, 28125 Universidade Federal do Rio de Janeiro, 21941-902 Rio de Janeiro, Brazil

## Abstract

Processing of biomass residues for material and energy recovery generate effluents containing complex organic mixtures. Analyses of bulk parameters are conventional for characterization and classification of such effluents, where the limited information offered impedes the development of molecular level management practices. This study assessed the potential of solvent-selective complexity reduction of effluent dissolved organic matter (DOM) from anaerobic bioprocessing of biomass residues for ^1^H NMR spectroscopy. The DOM were acquired after filtration and drying of samples from seven full scale anaerobic bioreactor facilities with effluents used as biofertilizer. The ^1^H NMR spectra of DOM in indigenous solvent (water) revealed source dependent characteristics primarily due to variable abundance of aliphatic CCH in lipids and peptides, OCCH in carbohydrates, and olefinic and aromatic subunits. Dimethyl sulfoxide solubilized larger proportion of nonfunctionalized aliphatic and aromatic molecules, with ^1^H NMR features also varying depending on the DOM source. Methanol, however, reduced the ^1^H NMR spectral variability and dissolved sets of aliphatic and aromatic molecules from the dried DOM with similar ^1^H NMR features irrespective of their origin. Among the other solvents studied, the reactive dissolution by trifluoroacetic acid decomposed aliphatic units while enriching aromatics (i.e., C_ar_ **H**:CC**H** of 0.7 compared to 0.2 in water), also forming small (oligo)­saccharides and peptide fragments. Acetone, dichloromethane, and acetonitrile extracted alkyl-rich molecules with varying degrees of functionalization. Acetonitrile separated a fraction enriched in aliphatic carboxylic acids, while dichloromethane mainly dissolved nonfunctionalized aliphatic hydrocarbons. It is proposed that a simple process of filtration, drying, and dissolution of effluent DOM in different solvents substantially reduces the complexity and heterogeneity of the organic mixtures enabling the structural discrimination of diverse molecular classes by ^1^H NMR spectroscopy.

## Graphical abstract

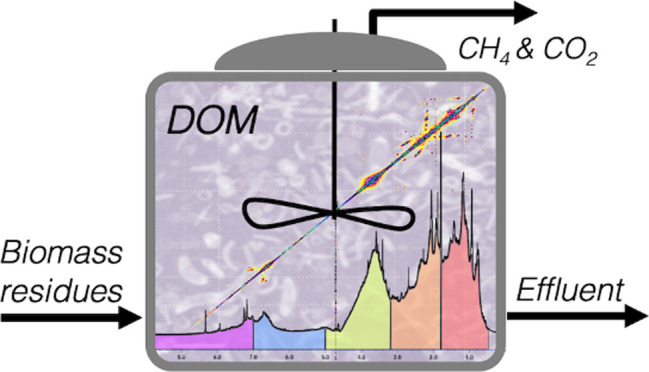

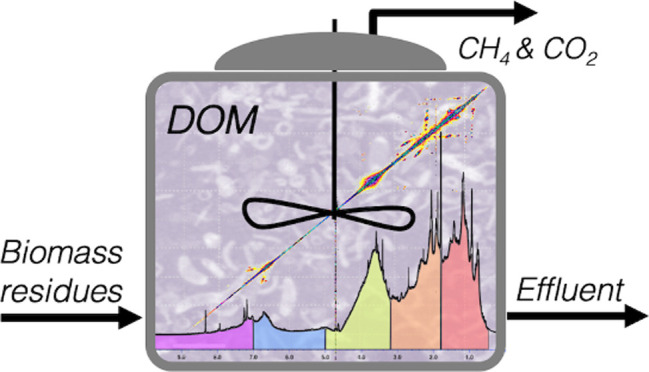

## Introduction

Processing of biomass residues such as livestock manure, crop residues, municipal and industrial wastes, and wastewaters, for volume reduction, materials and energy recovery generate effluents containing complex and heterogeneous mixtures of organic molecules. In particular, anaerobic bioprocessing are widely used for production of biomethane as a renewable energy carrier, and recovery of nutrients (i.e., C, N, and P) through the land application of the effluents.^1^ The anaerobic bioprocessing is driven by microbial trophic interactions, in which the hydrolytic bacteria decompose macromolecules into mono- and oligomers by extracellular enzymes such as cellulases, proteases, and lipases at early stages of processing.^2^ The products of microbial hydrolysis are further converted to simple molecules (i.e., methane, carbon dioxide, hydrogen, carboxylic acids, ammonia, and hydrogen sulfide) by fermentative and anaerobic organic acid-oxidizing bacteria and methanogenic archaea.^3^

Carbon conversion in anaerobic bioreactors is extensive across the molecular spectrum, as exemplified by organic matter reduction to methane in excess of 75% of input organic compounds in well performed processes.^4^ This implies drastic changes of both the chemical composition and bulk properties of input materials.^5^ While the main molecular characteristics of small effluent molecules are largely known,^6^ the complex mixture of organic molecules is ill-defined. Distinction between primary and secondary chemical structures is only partially addressed e.g., in relation to the characterization of extracellular polymeric substances and soluble microbial products.^7,8^ Empirical bulk parameters are conventionally used to characterize the feedstock and effluents based on elemental compositions (i.e., C, N, P, S contents), and for classification into categories based on e.g., chemical and biological oxygen demand, volatile solids (VS) determined by loss on ignition, carbohydrates, proteins, and lipids.^9,10^ Although the widespread application of bulk characterization has been proven practical, information loss is substantial due to the obscured molecular complexity.^11,12^

Processed and unprocessed biomass residues have heterogeneous matrices, where nontarget characterization is a viable option for exploring molecular heterogeneity and transformation mechanisms. Among the available options, nuclear magnetic resonance (NMR) spectroscopy exclusively offers isotope-specific quantification of atomic environments within the complex mixtures.^13^ The NMR data on isotopes such as ^1^H and ^13^C comprise chemical shifts (atomic environments), J-couplings (counts of neighboring atoms), line width (relaxation), and integrals (relative abundance), that can be used for quantification and chemical characterization of certain atomic environments in complex materials.^14^ NMR is nondestructive and allows combined analysis of complementary NMR spectra, well suitable for elucidating the molecular diversity in heterogeneous matrices,^15^ providing uncharted molecular resolution with the potential for developing molecular level management strategies and attaining a mechanistic understanding of the bioconversion processes,

Solid-state ^13^C cross-polarization magic angle spinning NMR spectroscopy provides the distribution of C among structural constituents of organic residues, including alkyl, O-alkyl, anomeric, aromatic, O,N-substituted aromatic, and carbonyl groups.^16,17^ The comparative assessment of the solution-state ^1^H–^13^C heteronuclear single quantum coherence (HSQC) NMR patterns of the influent and effluent organics distinguishes the chemical structures of labile fractions (e.g., olefinic bonds, and anomers with α linkages) from recalcitrant fractions (e.g., aromatics, lignin methoxyl, and anomers with β linkages) upon anaerobic bioprocessing.^5^

The ^1^H NMR spectroscopy is particularly favorable due to high sensitivity (isotopic abundance of ^1^H > 99.98%, and large gyromagnetic ratio) within manageable acquisition times.^13^ The one-dimensional ^1^H NMR spectrum combines information-rich quantification of nonexchangeable protons, when acquired with solvent-suppression.^18^ Already a visual inspection of line shape, relative section integrals, and clustering of ^1^H NMR resonances in certain spectral regions provides far-reaching leads about the molecular structures. Critically, complex materials with thousands of distinct molecules produce overlap of NMR resonances, resulting in NMR spectra with broad lines.

In this study, we explored the potential of solvent-selective complexity reduction of effluent dissolved organic matter (DOM) for structural characterization using ^1^H NMR spectroscopy. We acquired samples from commercial full scale anaerobic bioreactors with diverse feedstock profiles, a broad gradient of dissolved organic carbon (DOC) and from different geographical areas. In addition to providing insights on the organic matter transformation, understanding the molecular complexity of the DOM has a particular relevance due to the significance of DOM as a sustainable source of nitrogen and carbon.^6,12^ Since meaningful ^1^H NMR spectra could be acquired after drying and solubilization of DOM in deuterated solvents, samples were not further processed, e.g., by removing salts or possible (traces of) paramagnetic compounds, to assess distinction of molecular structures in indigenous matrices. The outcomes offer a simple concept with a broad utility for complexity reduction of effluent DOM and detailed structural elucidation of the organic constituents using ^1^H NMR spectroscopy.

## Experimental Section

### Sources of DOM

Samples were collected from effluent ports of seven full scale continuous stirred-tank anaerobic bioreactors (designated 3D, 4A, 5B, 6B, 7B, 7D, and 9C), located in Germany, Norway, Denmark, and Sweden. Main feedstock categories included municipal food waste, manure, agricultural residues, municipal sewage sludge, and energy crops, together with minor organic residues such as distillery waste, food industry waste, slaughterhouse waste, primary municipal sludge, surplus potatoes, sugar beet, wilted silage, and glycerol (Table 1). According to the information provided by the operators, the anaerobic bioreactors operated at temperatures between 38 and 51 °C, conventional to mesophilic and thermophilic anaerobic degradation processes, respectively. Organic loading rates, representing the rate of feedstock processing per volume of the bioreactor, spanned between 2.0 and 7.3 g VS l^–1^ d^–1^, and hydraulic retention times, representing the average time that the influent material resides inside the bioreactor, ranged from 20 to 42 days (Table S1). Sampling was performed in communication with the operators at each site according to the procedures used for routine monitoring, based on the technical specifications of each plant. Samples were transferred to the laboratory and filtered within 24 h (0.45 μm filter, VWR). The pH, total solid (TS), VS, DOC and carboxylic acids; acetic, propionic, butyric, iso-butyric, iso-valeric, n-valeric, iso-capronic, n-capronic, heptanoic acids, were measured. Sample properties and methods used for the measurements are detailed in Table S1. All samples were freeze-dried directly after filtering and stored in the dark at – 20 °C prior to the NMR spectroscopy.

**Table 1:** Temperature and Feedstock Profiles of the Anaerobic Bioreactors Used As the Source of Dissolved Organic Matter

Sample	T (°C)	Main feedstock fraction (% of total weight)
3D	38	Municipal food waste (84), distillery waste (10), slaughterhouse waste (5), mixture of grease separator and glycerol (1)
4A	40	Municipal food waste (52), cow manure (26), pig slurry (17), food industry slurry (5)
5B	40	Crops residue (80), food industry slurry (20)
6B	51	Cow manure (67), pig slurry (17), food industry slurry (8), energy crops (8)
7B	40	Cow manure (95), agricultural residue (5)
7D	38	Waste activated sludge (62), primary sludge (38)
9C	45	Energy crops (58), cow manure (32), mixture of potatoes, sugar beet, wilted silage (10)

### NMR Spectroscopy and Data Processing

Aliquots of the dried samples (30 – 45 mg) were dissolved in 0.75 mL of deuterated solvents in glass vials. Solvents used were deuterium oxide, methanol-d_4_, and DMSO-*d*_6_ (>99.96%D, Sigma-Aldrich). For a selected sample (i.e., 9C), acetic acid-d_4_ (99.9%D, Dr. Glaser AG Basel), trifluoroacetic acid-d (99.96%D, Sigma-Aldrich), acetone-*d*_6_ (99.96%D, Sigma-Aldrich), acetonitrile-d_3_ (99.95%D, Chemotrade), dichloromethane-d_2_ (100%D, ARMAR Chemicals), and pyridine-d_5_ (99.8%D, Merck) were also used. Extractions by solvents other than D_2_O were carried out uniformly under sonication for approximately 1 h at 40 °C, approximating the conventional operating temperatures of the anaerobic bioreactors.

The ^1^H NMR spectra were acquired using a Bruker Avance III HD 600 MHz spectrometer equipped with a 5 mm BBO/H&F/D cryoprobe (90° pulse ∼ 10 ms; SW­(^1^H) ∼ 20 ppm), at NMR core facility in Umeå, Sweden. The acquisition was done using solvent suppression (*noesypr1d*) with 5 s presaturation to attenuate the residual water signal, 1 ms mixing time, 5 s acquisition time, and 1 Hz line broadening. Spectral processing and analyses were performed by Bruker TopSpin 4.1.4. Principal component analysis (PCA) was conducted on centered spectral data normalized to a constant sum using Simca 13.0 (Umetrics, Sweden).^19^ The aim was to assess differences in NMR resonance amplitudes across the chemical shift range that determined sample variation from different sources. The PCA was performed on ^1^H NMR spectra from samples acquired in identical solvents to avoid biases caused by solvent induced displacement of chemical shifts and contribution of solvent impurities. Assignments to the chemical shift regions within the ^1^H NMR spectra were made based on literature data (see e.g., Hertkorn et al.,^20^ Simpson et al.,^21^ Chalbota and Kavouras^22^), and the acquisition of complementary two-dimensional NMR spectra for a selected sample (i.e., 260 mg of 9C sample in deuterium oxide). Parameters used for the acquisition of the two-dimensional NMR spectra are listed in Table S2. Briefly, J-resolved (JRES) and total correlation spectroscopy (TOCSY) were carried out to provide relationships between hydrogen atoms commonly 2 to 4 bonds apart together with multiplicity-edited ^1^H–^13^C distortionless enhancement by polarization transfer HSQC NMR spectroscopy to distinguish CH_2_ from CH and CH_3_ resonances.^23^

## Results and Discussion

### Overall Sample Properties

The pH values of effluents from seven anaerobic bioreactors ranged between 7.3 and 7.8. The TS (dry matter) varied from 4 to 9% of the total weight and VS contents (organic fraction) from 66 to 86% of the TS. After filtration, DOC concentrations ranged from 91 to 1868 mg C l^–1^, representing a broad concentration gradient of dissolved carbon (Table S1). Among the short chain carboxylic acids, only acetate was quantifiable in two samples (6B and 9C) with relatively low concentrations (<1% of DOC; Table S1). Instabilities in performance of anaerobic bioreactors are manifested in the form of carboxylic acids accumulation and low pH, representing kinetic imbalances in the cascade of microbial pathways responsible for organic matter decomposition to methane.^24^ A near-neutral pH and low concentrations or absence of short-chain carboxylic acids in the samples imply that the biodegradation sequence of hydrolysis, fermentation, anaerobic organic acids oxidation, and methanogenesis operated without apparent imbalances at the time of sampling.

### ^1^H NMR Features of Effluent DOM

The ^1^H NMR spectra of dried DOM from seven anaerobic bioreactors dissolved in D_2_O showed congruent envelopes of superimposed yet distinct resonances across the typical C_sp3_ **H** (0 – 5 ppm) and C_sp2_ **H** (5 – 10 ppm) chemical shifts (Figure S1). Representative ^1^H NMR features of DOM are depicted by an area-normalized sum of the ^1^H NMR spectra acquired from the seven anaerobic bioreactors (Figure 1). Five section integrals provide the relative contribution of core C**H** substructures, including nonfunctionalized aliphatic CC**H** (e.g., in lipids and peptides; δ_H_: 0.5 – 1.9 ppm), remotely oxygenated OCC**H** (e.g., in carboxyl-rich alicyclic molecules), together with NC**H** (e.g., in amides and amines; δ_H_: 1.9 – 3.1 ppm), directly oxygenated OC**H** (e.g., in ethers, esters and carbohydrates; δ_H_: 3.1 – 5.0 ppm), O_2_C**H** and =C**H** (e.g., in anomers, single and conjugated olefins, and phenols; δ_H_: 5.0 – 7.0 ppm), and C_ar_ **H** (e.g., mono- and polyaromatics and heterocycles; δ_H_: 7.0 – 10 ppm).^25^ The major ^1^H NMR resonances of DOM (in D_2_O) originate from CC**H**, with relative contributions of 33 – 43% of the total ^1^H NMR integrals in all samples (Table S3). All spectra contain near equal shares of OC**H** and OCC**H** resonances, with 24 – 27 and 23 – 28% of total integrals, respectively. The combined C_sp2_ **H** resonances at a chemical shift of 5 – 10 ppm (i.e., sum of O_2_C**H**, =C**H**, and C_ar_ **H**) comprise 6 – 17% of the total integrals (Table S3), corroborating predominance of saturated aliphatics over aromatics as a common feature of the DOM from anerobic bioreactors, which is apparently larger than those in natural DOM e.g. in freshwater and marine systems.^26^

**Figure 1:**
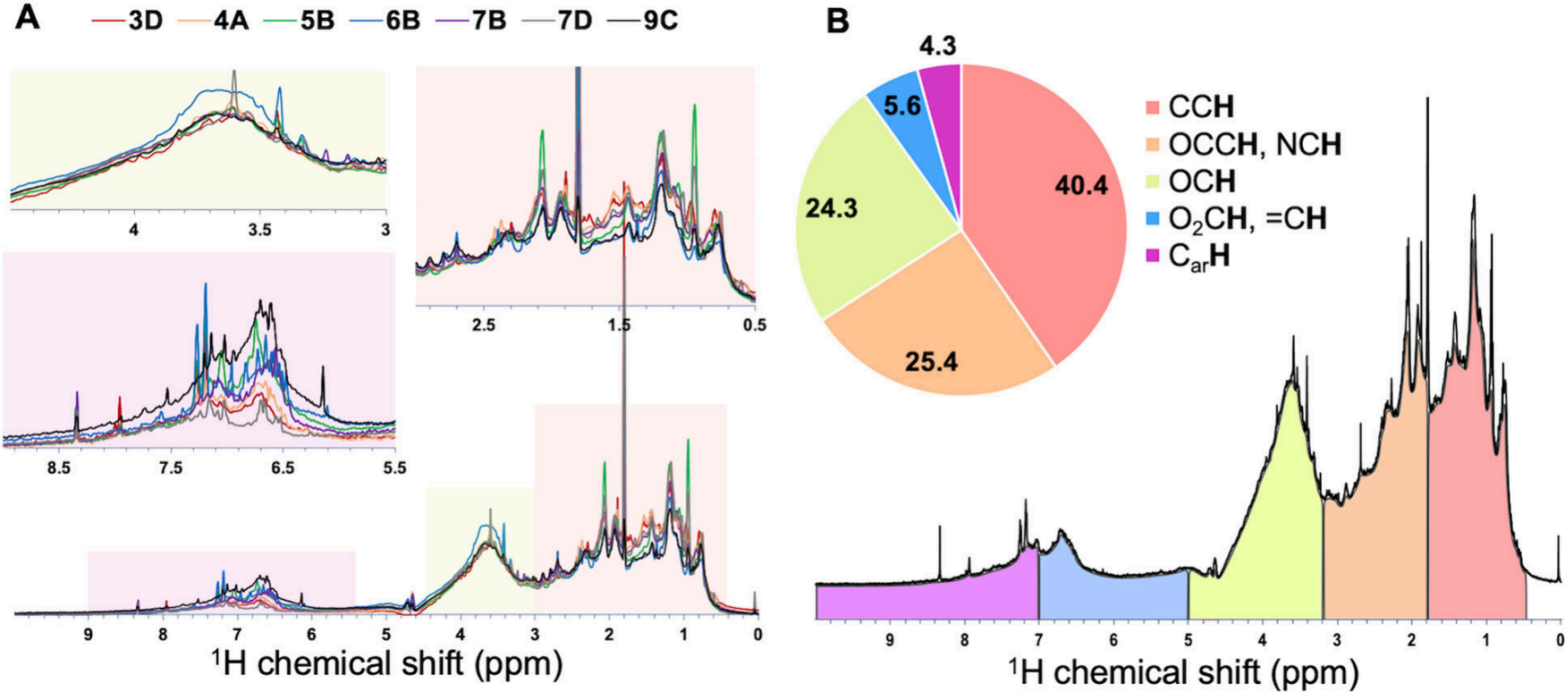
Area-normalized ^1^H NMR spectra (600 MHz) of effluent dissolved organic matter (DOM) from seven anaerobic bioreactors in D_2_O (A). Sum of ^1^H NMR spectra and averaged integral values of five spectral sections for the major organic subunits, presented as a pie chart (B). Data for individual samples are presented in Table S3. More detailed assignments of the NMR resonances are provided in the text.

### ^13^C–^1^H_123_ and ^1^H–^1^H Interconnections in Effluent DOM

^1^H NMR spectra of all samples show distinct signal envelopes between δ_H_: 0.5 – 0.9 and 0.9 – 1.0 ppm, representing methyl in alkyl groups (C**H**_3_) followed by the multiple resonances of methyl, methylene and methine units in branched alkyl and remotely oxygenated atomic environments between δ_H_: 1.0 – 1.9 ppm (Figure 1). Across the chemical shift region of δ_H_: 0.5 – 1.0 ppm, the JRES spectrum (acquired for 9C) predominantly contains doublets (J_HH_ ≤ 4 Hz), indicating the coupling of methyl with methine (i.e., interchain C**H**-C**H**_3_), and triplet cross peaks (J_HH_ ≤ 7 Hz) due to methyl connectivity to methylene units (i.e., terminal C**H**_2_-C**H**_3_; Figure 2). The multiplicity–edited ^1^H–^13^C HSQC NMR spectrum also confirms that the resonance envelopes between δ_H_: 0.5 – 1.0 ppm originate from multiple methyl groups with ^13^C chemical shifts in the span of δ_C_: 10 – 22 ppm (Figure 2). However, the CH_3_ cross peaks extend downfield beyond the typical chemical shift of δ_H_: 0.5 – 1.0 ppm to approximately 1.4 ppm. This feature, also observed for natural DOM, suggests the presence of methyl units close to carbonyls (C**H**_3_-C–COX), other remote oxygenation (**H**_3_CCCO units in structurally elaborate molecules) and/or positioned in branched alkyl chains (C**H**_3_-CC_n_).^27^ These CH_3_ resonances overlap with those of methylene and methine in one-dimensional ^1^H NMR spectra but can be distinguished in the HSQC NMR experiments. The abundance of CH-C**H**_3_ motifs across δ_H_: 0.5 – 1.4 ppm infers that the aliphatic moiety in DOM is substantially branched. The branching pattern is further evident by the multiple cross peaks in the TOCSY NMR spectrum at δ_H_:∼0.7 and ∼ 0.9 ppm that correlate with the downfield signals of methylene protons in the chemical shift span of δ_H_: 1.0 – 1.9 ppm (Figure 2).

**Figure 2:**
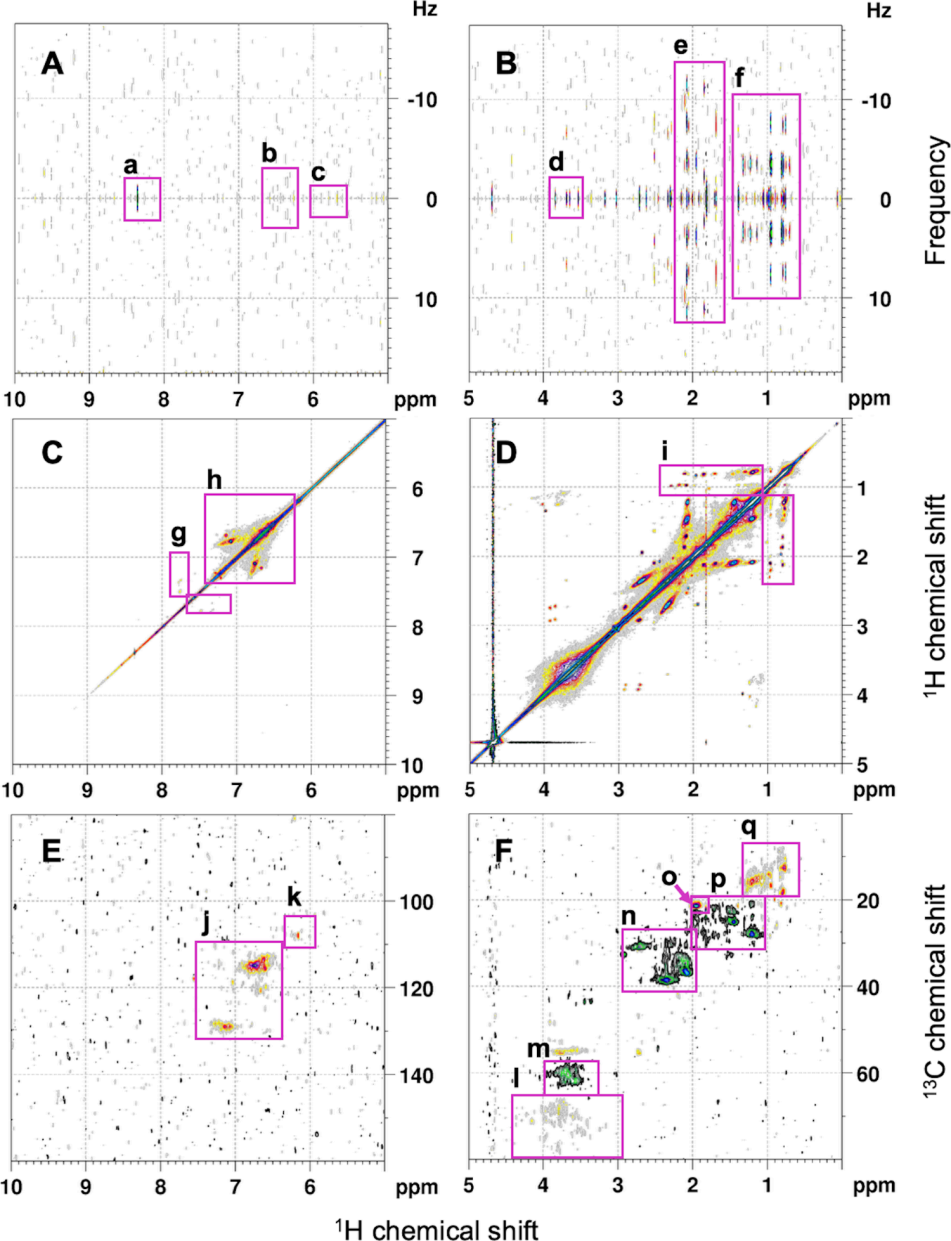
Complementary two-dimensional NMR spectra (600 MHz, D_2_O) acquired for effluent dissolved organic matter (DOM) from 9C. JRES NMR spectra (A and B); (a) formic acid, (b) olefinic protons with J_HH_ ≤ 3 Hz, (c) olefinic protons with J_HH_ ≤ 1 Hz, (d) methoxy singlets, (e) multiple methylene groups bound to methyl (quartets, J_HH_ ∼ 12 Hz), (f) methyl groups bound to methine (doublets, J_HH_ ≤ 4 Hz) and methylene (triplets, J_HH_ ≤ 7 Hz). TOCSY NMR spectra (C and D); (g) polycyclic aromatic protons, (h) CH–CH connectivity in monoaromatics with ortho and/or para oxygenated substituents, (i) methyl groups connected to methylene units. ^1^H–^13^C HSQC NMR spectra, CH_2_: black, green and blue, CH_3_ and CH: gray, yellow and red (E and F); (j) aromatic CH–CH units in two separate envelopes of monoaromatic protons at δ_H/C_ of 6.6 – 7.0/110 – 125 and polyaromatic protons at δ_H/C_ of 7.0 – 7.2/125 – 130 ppm, (k) olefinic CH=CH double bonds, (l) methoxy units, O–CH_3_, (m) methylene adjacent to oxygen, C–CH_2_–O, at δ_H/C_ of 3.5 – 4.0/55 – 65 ppm, (n) methylene close to oxygenated carbon units, C–CH_2_–COX, at δ_H/C_ of 2.0 – 3.0/30–40 ppm, including NCH_2_, (o) methyl groups connected to carboxyl derivatives, CH_3_–COO at δ_H/C_ of 1.95/21, (p) C–CH_2_–C at δ_H/C_ of 1.0 – 2.0/20 – 30 ppm, where purely aliphatic chains (i.e., [CH_2_]_n_) demonstrate a clear signal at δ_H/C_ of 1.2/28, (q) terminal methyl groups of aliphatic units (δ_H_ < 1.0 ppm) and extended C–CH_3_ signals (δ_H_:1.0 – 1.4 ppm) indicating the remote aliphatic branching.

Aliphatic methylene cross peaks (C–C**H**_2_–C) stretch downfield across the ^1^H chemical shift and form a distinct envelope in the HSQC NMR spectrum between δ_H/C_ 1.0 – 1.9/20 – 30 ppm (Figure 2). The OCC**H** units show several distinct resonances at δ_H_: 1.9 – 3.1 ppm attributable to remotely oxygenated CH units, where carbonyl derivatives **CH**_3_-COX show a strong resonance at δ_H/C_ of 1.95/21 ppm in the HSQC NMR spectrum, which may as well arise from acetic acid, **CH**_3_-COOH (Figure 2). Methylene units proximate to an oxygenated C, C-**CH**_2_-COX, forms an envelope in the HSQC NMR spectrum between δ_H/C_ 2.0 – 3.0/30 – 40 ppm, encompassing N**CH**_2_ methylene as well (intense peak at δ_H_: ∼ 2.7 ppm). Remotely oxygenated methylene groups particularly in the δ_H_: 2.0 – 2.2 ppm show multiple quartet cross peaks in the JRES spectrum, suggesting the coupling to methyl units and the presence of C**H**_3_-C**H**_2_-COX structures. Less pronounced resonances, commonly occurring between δ_H_: 2.2 – 3.1 ppm represent CH_n_ units in aliphatic carboxylic acids, and other remotely oxygenated units.^28^

Directly oxygenated OC**H** separates into two HSQC NMR regions attributable to methoxy units, O-**CH**_3_, with strong signals at δ_H/C_ 3.5 – 3.8/57 – 63 ppm (e.g., from methyl esters, methyl ethers, and carbohydrates) and methylene from multiple small molecules, C-**CH**_2_-O, at δ_H/C_ of 3.5 – 4.0/55 – 65 ppm (e.g., alcohols, ether, lactone, and ester derivatives). Further downfield, a small peak at δ_H_: 6.15 ppm indicates the presence of olefinic C**H**=C**H** units, corroborated by isolated cross peaks in both JRES and HSQC NMR spectra (Figure 2). The predominant resonance in the chemical shift of δ_H_: 6.2 – 7.0 ppm originates from the aromatics with oxygenated substituents (e.g., phenol derivatives; C_ar_ **H**-OX). Four major peaks appear at δ_H_: 7.15 – 7.22, 7.22 – 7.30, 7.92 – 7.95 and 8.30 – 8.35 ppm. The peaks at chemical shifts of δ_H_: 7.0 – 7.3 ppm are from alkylated and nonsubstituted monoaromatics, whereas peaks at δ_H_ > 7.3 ppm are from carboxylated benzene derivatives, fused benzene geometries, and probably six-membered N-heteroaromatics. In the JRES spectrum, no notable C_sp2_ **H** features can be observed, except for a singlet at chemical shift of δ_H_: ∼ 8.3 ppm, likely from formic acid.^28^ The TOCSY and HSQC NMR spectra demonstrate typical cross peaks of the C**H** in monoaromatic units with ortho and/or para oxygenated substitution as well as the cross peaks of polycyclic aromatics.

### CD_3_OD and DMSO-*d*_6_ Selective DOM

The dried DOM samples were readily solubilized in D_2_O, while they were only partially soluble in CD_3_OD and DMSO-*d*_6_. The sum of ^1^H NMR spectra acquired from the dried DOM samples solubilized in DMSO-*d*_6_ and CD_3_OD is presented in Figure 3 and their individual spectra in Figure S2. For CD_3_OD and DMSO-*d*_6_ extracts, the ^1^H NMR spectra were populated across chemical shift regions similar to those for DOM in D_2_O, yet with higher proportion of CC**H** resonances and a lower proportion of OC**H**. On the other hand, the relative contributions of C_ar_ **H** were lower in CD_3_OD and higher in DMSO-*d*_6_ in comparison with D_2_O, due to the abundance of nonsubstituted aromatics (i.e., δ_H_: 7.0 – 7.3 ppm). Overall, the CD_3_OD and DMSO-*d*_6_ soluble fractions of DOM are enriched in alkyl and less functionalized units, especially evident for the spectrum of the DMSO-*d*_6_ extracts, where in particular the ^1^H resonances of phenol derivatives (C_ar_ **H**-OX) at δ_H_: 6.2 – 7.0 ppm have substantially lower intensities as compared to both D_2_O and CD_3_OD. Apparently, the insoluble residues retain a higher proportion of oxygenated subunits, which commonly mediate and favor water solubility.^29^

**Figure 3:**
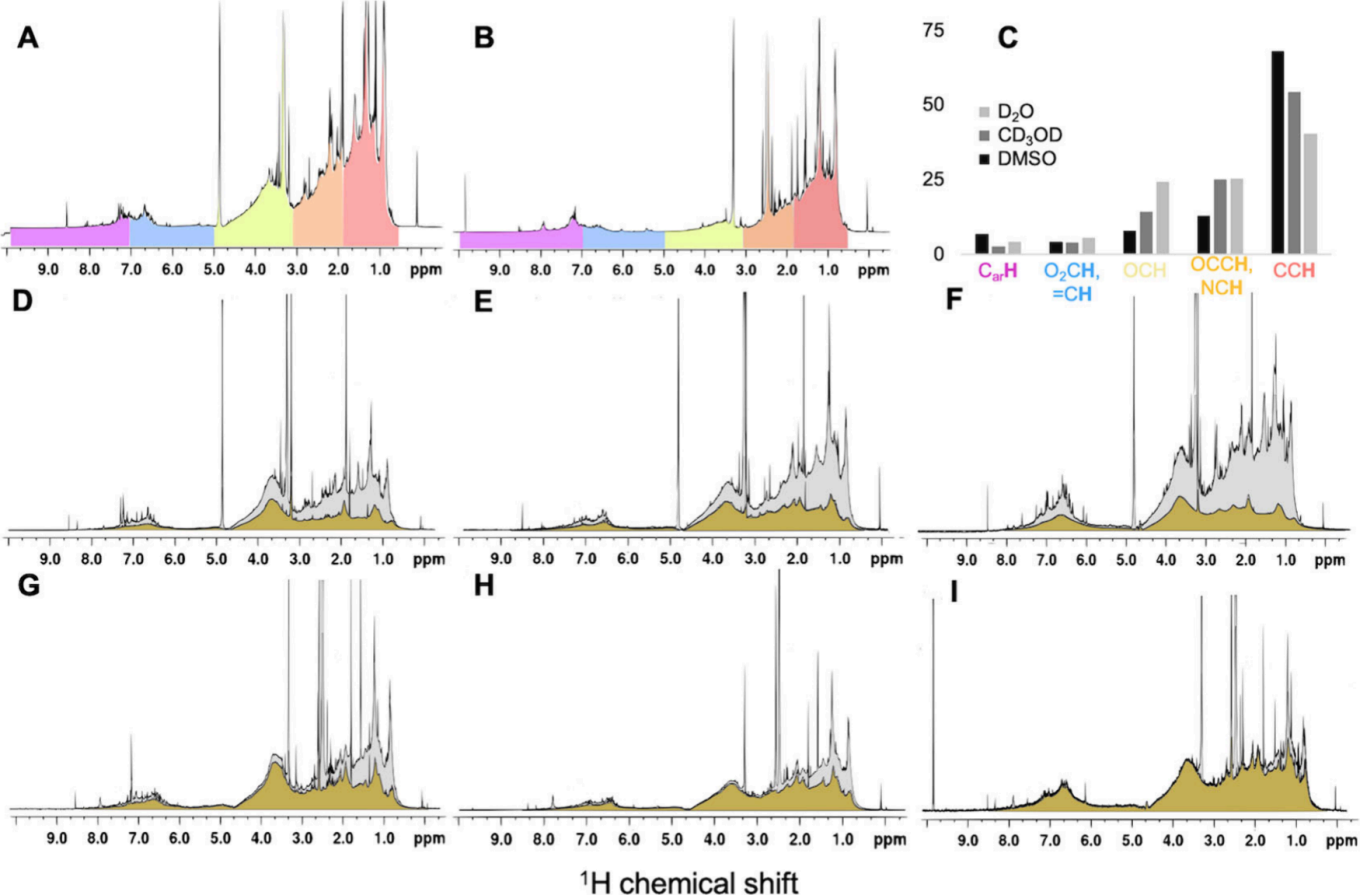
Sum of ^1^H NMR spectra of CD_3_OD extracted fraction (A) and DMSO-*d*_6_ extracted fraction of effluent dissolved organic matter (DOM) from seven anaerobic bioreactors (B). Comparison of the section integrals of the sum of the ^1^H NMR spectra of the DOM in D_2_O, CD_3_OD, and DMSO-*d*_6_ (C). Stacked ^1^H NMR spectra of the CD_3_OD-extracted fraction (gray) and the residual fraction solubilized in D_2_O (brown) for 6B (D), 7B (E), and 9C (F). Stacked ^1^H NMR spectra of the DMSO-*d*_6_ extracted fraction (gray) and the residual fraction solubilized in D_2_O (brown) for 6B (G), 7B (H), and 9C (I).

The remaining pellets after dissolution by CD_3_OD and DMSO-*d*_6_ were centrifuged and dissolved in D_2_O for selected samples (6B, 7B, and 9C). The spectra from residual fraction of DOM after CD_3_OD extraction lack sharp ^1^H NMR resonances with very few distinguishable peaks across both C_sp3_ **H** (0 – 5 ppm) and C_sp2_ **H** (5 – 10 ppm) chemical shifts (Figure 3). In other words, CD_3_OD separates highly abundant aliphatic and aromatic structures with lesser average oxygenation from a diverse set of low-abundance molecules that contribute to the underlying spectral background. Similarly, a large proportion of unresolved background ^1^H NMR resonances are missing in DMSO-*d*_6_ extracts that are particularly devoid of the OC**H** units compared to the ^1^H NMR spectra of DOM in D_2_O.

The experiment was complemented by a sequential solubilization of DOM in DMSO-*d*_6_→CD_3_OD→D_2_O, and CD_3_OD→DMSO-*d*_6_→D_2_O to further assess the selectivity of the solvents toward specific structural units (Figure S3). Through each step, the pellets were separated by centrifugation before adding the next solvent. The solubility of the DOM in DMSO-*d*_6_ was reduced after initial solubilization in the CD_3_OD, as evidenced by the low signal-to-noise ratio of the ^1^H NMR spectrum (Figure S3). The alkyl CH_n_ resonances in the region of δ_H_: 0.5 – 1.3 ppm are the remaining key signals in the ^1^H NMR spectra of the DMSO-*d*_6_ extracts following initial dissolution in CD_3_OD along with a broad aromatic resonance. However, the CD_3_OD-soluble fraction of the DOM was only subtly altered by the earlier, low yield, DMSO-*d*_6_ extraction. The spectra obtained from the CD_3_OD extracts following their prior dissolution by DMSO-*d*_6_ were almost identical to those obtained from DOM directly dissolved in CD_3_OD (Figure S3).

### Variations among the ^1^H NMR Spectra

The PCA was performed separately on the sets of samples recorded in D_2_O, CD_3_OD and DMSO-*d*_6_. Despite the similarities in line-shapes, the PCA revealed noticeable variations of ^1^H NMR features of DOM from different sources when dissolved in D_2_O (Figure S4). Assessment of the first loading plot (capturing 45% of spectral variances) shows that dissimilarities largely arise from differences in the abundances of aliphatic CC**H** (δ_H_: 0.5 – 1.9 ppm) and the OCC**H** resonances (δ_H_: 1.9 – 2.5 ppm) together with C_ar_ **H** of aromatic protons, with some preference of polyphenols over other C_ar_ **H** units (δ_H_: 6.5 – 7.5 ppm). Large variations are also observed among the ^1^H NMR spectra from the DMSO-*d*_6_ extractable fractions. The PCA first loading plot (capturing 42% of spectral variance) attributed the differences to a wide range of aliphatic CC**H** signals (δ_H_: 0.5 – 1.9 ppm) and different abundances of nonsubstituted aromatics (δ_H_: 7.0 – 7.3 ppm; Figure S4), a notable contrast to samples dissolved in D_2_O and CD_3_OD.

Notably, a few aliphatic resonances at δ_H_: 0.5 – 0.9 ppm (C**H**_3_) and δ_H_: 1.0 – 1.5 ppm (C–C**H**_2_-C) contributed to spectral dissimilarities among the samples in the PCA first loading plot (capturing 72% of spectral variances), when CD_3_OD was used as the solvent (Figure S4). Accordingly, dissolution of dried DOM from seven different anaerobic bioreactors in CD_3_OD separates a fraction with comparable ^1^H NMR features, suggesting that this solvent fractionates DOM compounds with similar structural properties across anaerobic bioreactors studied.

### Fractionation of Effluent DOM by Other Solvents

Aliquots of the dried DOM from one sample (i.e., 9C) were dissolved in solvents with a variety of dielectric constants, dipole moments, and functional groups (Table S4). To compare the ^1^H NMR spectral features of the fractions solubilized by different solvents, ^1^H NMR section integrals, methyl and methylene ratios (i.e., C**H**_3_:C**H**_2_), nonfunctionalized and functionalized C**H** ratios (i.e., CC**H**:O,N–C**H**), and aromatic and aliphatic C**H** ratios (i.e., C_ar_ **H**:CC**H**) were compared (Figure 4). Except for the reactive dissolution in trifluoroacetic acid, the DOM was only partially soluble in the solvents used. The degree of DOM dissolution in the solvents based on the contribution of the ^1^H resonances of the DOM to the total spectral resonances (i.e., including those from the solvent impurities) is in the order of methanol-d_4_ > DMSO-*d*_6_ ≈ acetic acid-d_4_ > dichloromethane-d_2_ > acetone-*d*_6_ > acetonitrile-d_3_ > pyridine-d_5_ (Table S5). The ^1^H NMR spectrum of the acetic acid (CD_3_CO_2_D) extract resembled those observed for DOM in D_2_O with a CC**H**:O,N–C**H** ratio of 1.0, C_ar_ **H**:CC**H** ratio of ∼ 0.2 (Figure 4), and we observed no distinctive feature except for the anticipated downfield resonance of the combined exchangeable protons (e.g., O**H**, CON**H**, N**H** and COO**H** groups) at δ_H_ ∼ 11.5 ppm (Figure S5).

**Figure 4:**
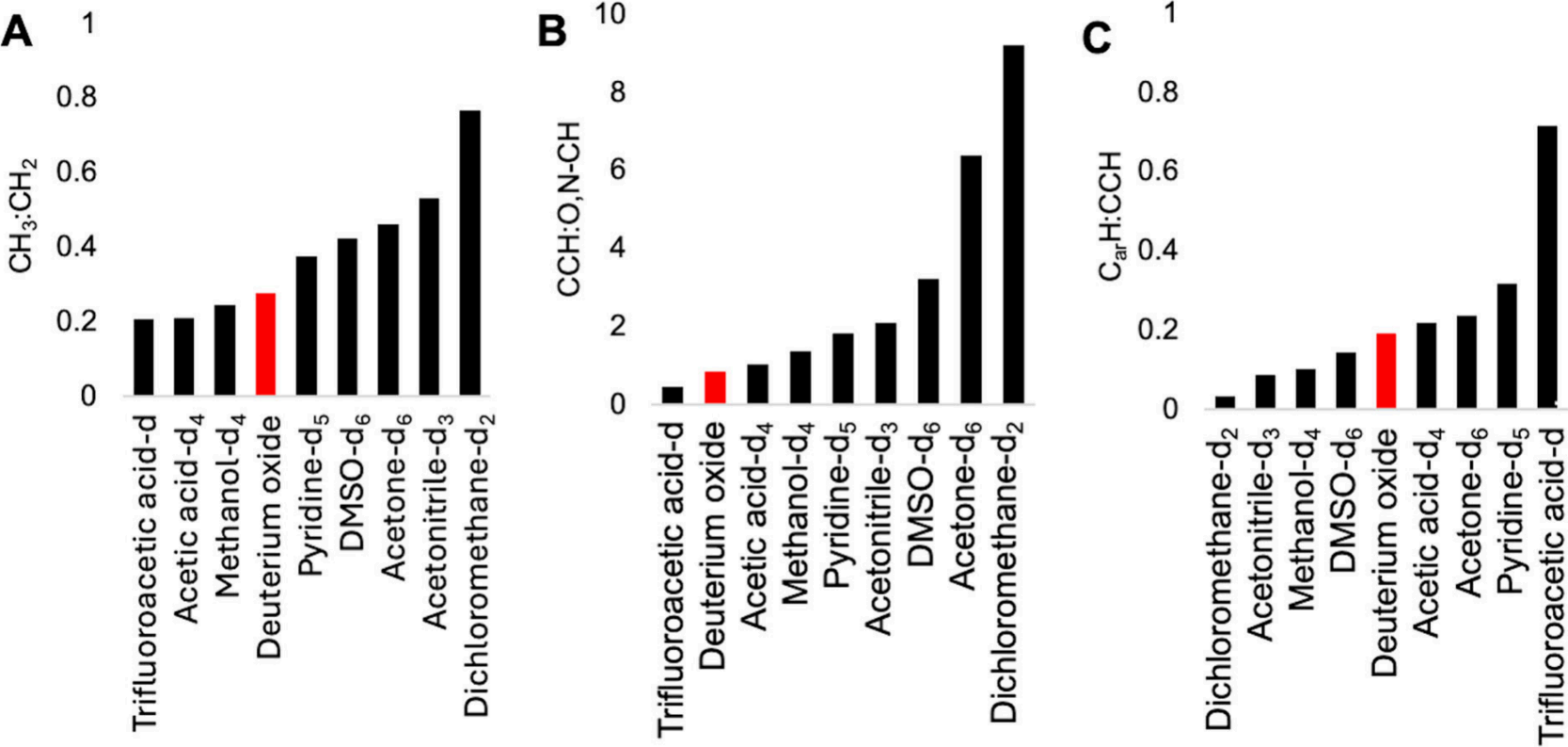
Changes in CH_3_:CH_2_ (A), CCH:O,N–CH (B), and C_ar_H:CCH ratios (C), computed from the ^1^H NMR spectra of the dried dissolved organic matter (DOM) in different solvents. Spectral integrals for different chemical shift regions are presented in Table S5.

Trifluoroacetic acid (CF_3_CO_2_D) also combines downfield ^1^H NMR resonances from exchangeable protons (δ_H_: ∼ 11.6 ppm) (Figure S5). This strong acid is highly reactive and traditionally used for the hydrolysis of organics prior to the compositional analyses of their structural constituents.^30^ The dissolution of DOM in trifluoroacetic acid was accompanied by evolution of gas and generation of heat. Upon spectral processing, referencing of δ_H_ was not straightforward and was done by alignment of its methyl (δ_H_: ∼ 0.7 ppm) and methylene (δ_H_: ∼ 1.2 ppm) ^1^H NMR resonances. The reaction eliminated mainly aliphatic C**H**_3_ and C**H**_2_ units, resulting in the lowest CC**H**:O,N–C**H** ratio of 0.45, while aromatics were enriched with a C_ar_ **H**:CC**H** ratio of 0.7 as the highest value among the solvents (Figure 4), even though the phenolic resonances (δ_H_ < 7.0 ppm) were attenuated in comparison with other C_ar_ **H** units (Figure S5). The enrichment of a multitude of ortho-/para-substituted aromatics and distinct polyaromatic C**H**-C**H** are particularly visible when acquiring a complementary TOCSY NMR spectrum of the trifluoroacetic acid extract at δ_H_: 6.5 – 9.0 ppm (detailed in Figure S6). A distinctive feature of the trifluoroacetic acid extract is the emergence of downfield OC**H** (δ_H_: 4.3 – 4.8 ppm) and O_2_C**H** resonances (δ_H_: 5.0 – 6.0 ppm; Figure S5). Although the ^1^H NMR signals in this region are unresolved, a few high intensity TOCSY cross peaks at δ_H/H_ of 5.4/4.6, 5.6/4.5, 5.6/4.7, and 5.7/4.8 ppm indicate the correlation of diverse anomeric O_2_C**H** with OC**H** protons (detailed in Figure S6). Cross peaks were also observed in the chemical shift region where CON**H**-C**H** correlate at δ_H/H_ of 1.6 – 3.3/6.6 – 6.8 and at δ_H/H_: 6.7/1.8 ppm (Figure S6). These observations suggest the decomposition of polysaccharides to a suite of small (oligo)­saccharides as well as formation/enrichment of peptides fragments upon reaction of the DOM with trifluoroacetic acid.

Acetone (CD_3_COCD_3_) dissolved a distinct set of primarily alkyls with C**H**_3_:C**H**_2_ ratio of 0.5 (Figure 4) and a few small molecules. An unresolved splitting pattern was observed between δ_H_: 0.8 and 0.9 ppm followed by triplets of the terminal CH_2_–C**H**_3_ (Figure S5). Immediately downfield, two sharp peaks resonate at δ_H_: 1.05 and 1.10 ppm, resembling the deshielded C**H**_3_, likely due to the presence at the ß-position of heteroatoms (commonly shifting downfield from δ_H_: 0.9 ppm by 0.2 to 0.6 ppm). Two strong singlets at δ_H_: 2.8 and 3.3 ppm possibly indicate NC**H** and NC**H**_2_ units (Figure S5). In addition, DOM dissolution in acetone resolved patterns of coupled spin systems obscured in the ^1^H NMR spectrum of DOM in D_2_O (Figure S5). Between δ_H_: 4.15 to 4.25 ppm (OC**H** region), a spin–spin coupling with 8 lines emerged, which is the characteristic of the AB protons of an ABX spin system, combining 4 doublets with the same separation (i.e., J_AB_: 11 Hz).^31^ The share of aromatic compounds (16% of total ^1^H NMR integrals) is attributable to two multiplets at δ_H_: 7.6 and 7.8 ppm that probably indicate ortho-dicarbonyl derivatives e.g. phthalates or aromatic carboxylic acids (Figure S5).

The ^1^H NMR spectrum of DOM in acetonitrile (CD_3_CN) showed low signal-to-noise ratio of DOM resonances with even more selectivity toward branched alkyl groups than observed in acetone-*d*_6_ extract. Sharp resonances between δ_H_: 0.8 and 0.9 ppm represented superposition of many branched alkyl groups. Downfield, at δ_H_: 1.27 ppm, broad resonances of branched alkyl ^1^H NMR signals with a characteristic tailing pattern toward higher δ_H_ are visible (Figure S5), appearing as TOCSY cross peaks with methyl, at δ_H_: < 1 ppm, and other aliphatic CC**H** units at δ_H_: 1.6 ppm (detailed in Figure S7). The resonances between δ_H_: 1.1 and 1.4 ppm show a high-order splitting pattern of A_2_B_2_ spin systems in the ^1^H NMR spectrum,^31^ representing the combined magnetically equivalent four-hydrogen of many different aliphatic -C**H**_2_-C**H**_2_- branches (Figure S5). A broad resonance at δ_H_: 4.64 ppm (i.e., OC**H** region) is indicative of alkyl CH_2_ proximate to oxygen. Altogether, the fraction solubilized by the acetonitrile encompasses the ^1^H NMR spectral feature of the aliphatic carboxylic acids. We commonly observe two strong symmetric broadened triplets between 5.2 and 6.4 ppm typical for acetonitrile-d_3_ and observed repeatedly in other DOM samples previously (explanation is yet unclear but may include participation of exchangeable protons).

DOM in dichloromethane (CD_2_Cl_2_) showed 87% of the ^1^H NMR integrals from deeply branched alkyl units (δ_H_: 0.5 – 1.9 ppm) and considerable methyl content, with highest C**H**_3_:C**H**_2_ ratio of 0.8 as well as the highest CC**H**:O,N–C**H** ratio of 9.2 compared to the other solvents (Figure 4). The sharp ^1^H NMR resonances at δ_H_: 1.2 and 1.4 ppm nevertheless represent deeply overlapping alkyl species, with very low contributions from oxygenated aliphatic units (OC**H**). Carboxylic acids are present and HOOC-C**H**- resonates from δ_H_: 2.0 – 2.5 ppm. Diverse but very marginal resonances appear in the chemical shift regions of OCC**H** (δ_H_: 2.2 – 3.0 ppm), OC**H** (δ_H_: 3.0 – 4.0 ppm), and aromatics C_ar_ **H** units (Figure S5). Accordingly, unsubstituted, branched aliphatic hydrocarbons are largely abundant in dichloromethane than the substituted and aromatics units. Strong intra-alkyl C**H**_n_-C**H**_n_ cross peaks dominate the TOCSY NMR spectrum of the dichloromethane extract, further corroborating the selectivity of this solvent to dissolve the unsubstituted aliphatic hydrocarbon fraction of effluent DOM (detailed in Figure S7).

Pyridine (C_5_D_5_N) dissolved a considerably wider range of aliphatic molecules than acetone and dichloromethane, with CC**H** units contributing 54% of total ^1^H NMR integrals. J-couplings are not resolved, and an inconspicuous tailing resonance carries relevant ^1^H NMR integral down to 4.7 ppm. The poor resolution of individual ^1^H NMR resonances in pyridine extracts testifies for a huge diversity of low abundance atomic environments and considerable proportions of remotely oxygenated molecules (OCC**H** units), and lesser contributions from OC**H** units (Figure S5). Aromatic hydrogen atoms (C_ar_ **H**) are unaccountable because of overlap of C_5_ **H**D_4_N resonances, except for a peak at 7.9 ppm probably from formic acid, with a relative abundance of 4.3% of ^1^H NMR integrals.

## Conclusions

^1^H NMR spectroscopy of effluent DOM from seven full scale anaerobic bioreactors processing diverse mixtures of organic residues detected recognizable variations in the abundances of aliphatic CCH (e.g., in lipids and peptides), OCCH (e.g., carboxyl-rich alicyclic molecules), and aromatic subunits (e.g., mono- and polyaromatics and heterocycles). The aliphatic moieties appeared substantially branched, as evidenced in the HSQC NMR spectrum due to the downfield extension of the CH_3_ signals beyond their typical chemical shift range. Partial dissolution of dried DOM in deuterated solvents other than D_2_O enabled solvent-selective complexity reduction of the DOM into mixtures with distinctive ^1^H NMR features. In particular, methanol extracted highly abundant aliphatic and aromatic structures with a high degree of similarity among the samples of different origin, suggesting that this solvent separates common metabolites and degradation products of the anaerobic degradation.

Dissolving the dried DOM in solvents with different properties revealed several interesting observations. The reactive dissolution of the dried DOM by trifluoroacetic acid removed the aliphatic CH_2_ and CH_3_ units, while enriching the aromatic, (oligo)­saccharide, and peptide constituents. Acetone demonstrated affinity toward CH units from protein and peptide fragments, acetonitrile favored aliphatic carboxylic acids, whereas dichloromethane fractionated nonfunctionalized, branched aliphatic hydrocarbons. These findings indicate that the ^1^H NMR spectroscopy combined with a simple process of filtration, drying and dissolution of DOM in different solvents allows acquiring uncharted structural information, including distinctive classes of molecules and even initially obscured spin coupling patterns. This offers an enhanced resolution for discerning the molecular complexity of effluents upon downstream handling of heterogeneous organic residues, as a step toward filling in the current analytical gap needed for characterizing these bioresources based on their chemical units rather than bulk properties.

## Supplementary Material



## Notes

The overall sample properties and their measurement methods; parameters used for acquisition of the complementary two-dimensional NMR spectra; integrals of ^1^H NMR spectra acquired for effluent DOM from seven samples in D_2_O, CD_3_OD, and DMSO-*d*_6_; properties of solvents used; integrals of ^1^H NMR spectra of DOM from 9C dissolved in different solvents; ^1^H NMR spectra of DOM from seven anaerobic bioreactors in D_2_O; ^1^H NMR spectra of CD_3_OD and DMSO-*d*_6_ extracted fraction in samples from seven anaerobic bioreactors; comparison of ^1^H NMR spectra DOM from 9C after sequential extraction; principal component analysis and loading plots of seven samples dissolved in D_2_O, CD_3_OD, and DMSO-*d*_6_; ^1^H NMR spectra of DOM from 9C dissolved in different solvents; complementary TOCSY NMR spectra of DOM from 9C dissolved in solvents other than D_2_O (PDF)
