# Supplementary material for: Solvent-Selective Complexity Reduction of Effluent Dissolved Organic Matter for 1H NMR Spectroscopy
Source: Anal Chem. 2026 Apr 7;98(15):11149–57. doi: 10.1021/acs.analchem.5c07560 (PMC13103931; doi:10.1021/acs.analchem.5c07560)
Supplement: Supplementary file 1 [file ac5c07560_si_001.pdf]

Supporting Information

Solvent-selective complexity reduction of effluent dissolved organic matter for ^{1}H NMR spectroscopy

Sepehr Shakeri Yekta[*,1,2], *Alex Enrich Prast*[1,2], *Mattias Hedenström*[3], *Tobias Sparrman*[3], *Luka Šafarič*[1,2], *Giacomo Carraro*[1,2], *Helena Rodrigues Oliveira*[4], *Thuane Mendes Anacleto*[5], *Annika Björn*[1,2], *Norbert Hertkorn*[1,2].

[1]Department of Thematic Studies - Environmental Change, Linköping University, 581 83 Linköping, Sweden. [2]Biogas Solutions Research Center, Linköping University, 581 83 Linköping, Sweden. [3]Department of Chemistry, Umeå University, 901 87 Umeå, Sweden. [4] Centro Federal de Educação Tecnológica Celso Suckow da Fonseca (CEFET/RJ), 20271 110 Rio de Janeiro, Brazil. [5] Unidade Multiusuário de Análises Ambientais, Instituto de Biologia, Universidade Federal do Rio de Janeiro, 21941-902 Rio de Janeiro, Brazil.

*Corresponding author: email: sepehr.shakeri.yekta@liu.se

Table of contents

Table S1 -- S3

Table S2 -- S4

Table S3 -- S5

Table S4 -- S6

Table S5 -- S7

Figure S1 -- S8

Figure S2 -- S9

Figure S3 -- S10

Figure S4 -- S11

Figure S5 -- S12

Figure S6 -- S13

Figure S7 -- S14

Table S1. Organic loading rate (OLR) and hydraulic retention time (HRT) of the full-scale anaerobic bioreactors provided by the operators as well as the overall sample properties and methods for the measurements.

Sample ID	OLR ($gVS^{-1} d^{-1}$)	HRT (days)	[a]pH	[b]TS (% of total weight)	[b]VS (% of TS)	[c]DOC ($mg\ C\ l^{-1}$)	[d]Acetic acid, C_2 ($mg\ C\ l^{-1}$)	[d]SCCA, C_3 - C_7 ($mg\ C\ l^{-1}$)
3D	4.0	30	7.5±0.0	4.1±0.0	68±0	654±15	<16	n.d.
4A	2.4	33	7.8±0.0	4.7±0.0	68±1	446±33	<16	n.d.
5B	n.a.	n.a.	7.3±0.0	8.7±0.5	86±1	511±3	<16	n.d.
6B	n.a.	n.a.	7.8±0.0	7.7±0.1	74±1	1868±36	25±3	n.d.
7B	n.a.	30	7.7±0.0	5.3±0.2	75±2	1021±96	<16	n.d.
7D	2.0	20	7.4±0.0	4.0±0.1	66±0	91±8	n.d.	n.d.
9C	7.3	n.a.	7.7±0.0	7.5±0.2	80±0	1646±16	19±2	n.d.

a) The pH was measured using a pH meter (InoLab 7310, WTW, Weilheim, Germany) according to the European standard method (EN12176:1998)

b) The total solid (TS) and volatile solid (VS) contents were measured weekly according to the Swedish Standard method SS-EN 12880.

c) Dissolved organic carbon (DOC) concentrations were analyzed using a Total Organic Carbon analyzer (Shimadzu TOC-VCSH, Japan) coupled to Non-Dispersive Infra-Red (NDIR) detector.

d) The concentration of short chain carboxylic acids (SCCA), including acetate, propionate, butyrate, iso-butyrate, valerate, iso-valerate, caproate, and iso-caproate, were quantified using a gas chromatograph (6890 Series, Hewlett Packard, USA) with a flame ionization detector (6890 Series, Hewlett Packard, USA) following the method by Jonsson and Borén. J. Chromatogr. A 2002, 963 (1–2), 393–400. The quantification limit of the analyses was 16 mg $C\ l^{-1}$.

Table S2. Parameters used for acquisition of the two-dimensional NMR spectra.

**Spectrum**	**Pulse program**	**Size of fid**	**DS/ NS/TD0**	**AQ (sec)**	**O1 (Hz)**	**LB (Hz)**	**Ph_mod**	**SR (Hz)**
^{1}H-^{1}H J-RES	jresgpprqf	F2: 17232, F1: 48	16/48/8	F2:1, F1:1.37	2821.66	F2:1, F1:3	F2: no, F1: mc	F2: 10.72, F1: 2820.78
^{1}H-^{1}H TOCSY	dipsi2gpphprzs	F2: 24036, F1: 1024	16/8/1	F2:1.25, F1:0.07	2821.66	F2:1, F1:0.3	F2: pk, F1: pk	F2: 10.92, F1: 0
^{1}H-^{13}C DEPT HSQC NMR	hsqcedetgpsisp2.3	F2: 4506, F1: 1024	8/64/1	F2:0.25, F1:0.02	2821.66 (^{1}H), 12073 (^{13}C)	F2:3, F1:6	F2: pk, F1: pk	F2: 9.55, F1: -1397.58

a) Abbreviations and parameters: JRES: J-resolved, TOCSY: Total correlation spectroscopy, DEPT: distortionless enhancement by polarization transfer, DS: Number of dummy scans, NS: number of scans, td0: Loop count for initial feed size, AQ: acquisition time, O1: transmitter frequency offset, LB: Line broadening, Ph_mod: phasing mode, and SR: spectrum reference frequency.

Table S3. Integrals of ^{1}H NMR spectra (% of total integrals) acquired for dissolved organic matter from seven samples in D_2O, CD_3OD and DMSO-d_6. Spectra were acquired with 1024 scans.

[a] Core CH substructures (chemical shift)	Solvent	3D	4A	5B	6B	7B	7D	9C
$C_{ar}H$ (7.0 - 10.0 ppm)	D_2O	4.1	3.3	4.5	4.3	5.8	3.8	6.7
	CD_3OD	2.8	3.2	0.9	2.6	3.2	3.6	2.1
	DMSO-d_6	7.6	5.5	12.8	4.8	6.6	3.4	15.3
O_2CH, CH=CH (5.0 - 7.0 ppm)	D_2O	5.0	2.9	7.0	7.0	6.9	4.1	10.6
	CD_3OD	2.4	4.4	1.3	5.0	3.7	3.3	6.7
	DMSO-d_6	5.4	3.7	4.0	5.0	4.7	1.8	4.9
OCH (3.1 - 5.0 ppm)	D_2O	23.9	25.0	22.8	28.2	24.6	24.5	24.6
	CD_3OD	17.3	18.1	5.2	17.9	12.1	15.4	16.7
	DMSO-d_6	12.4	8.9	5.0	8.7	5.2	5.3	3.0
OCCH, NCH (1.9 - 3.1 ppm)	D_2O	26.6	25.9	24.0	23.7	24.6	25.2	25.5
	CD_3OD	30.2	28.1	18.2	27.2	23.6	22.8	29.7
	DMSO-d_6	18.7	16.3	12.6	14.3	11.7	10.5	8.1
CCCH (0.5 - 1.9 ppm)	D_2O	40.4	42.9	41.6	36.9	38.2	42.4	32.6
	CD_3OD	47.3	46.2	74.4	47.4	57.4	54.9	44.9
	DMSO-d_6	56.0	65.6	65.8	67.2	71.7	78.9	68.7

a) ^{1}H chemical shift regions represent core CH substructures including, non-functionalized CCH, remotely oxygenated OCCH, encompassing also NCH, directly oxygenated, OCH, O_2CH and double bonds CH=CH, and aromatic H.

Table S4. Overall properties of solvents used to assess potential fractionation of the DOM. Information on solvents is obtained from https://acsgcipr.org/tools/solvent-tool/

Solvent	Chemical formula	Dielectric constant	Dipole moment	Functional groups	Properties	^{1}H Chemical shift of impurities (ppm)
Acetic acid-d_4	CD_3CO_2D	6.2	1.68	Acids	Protic	2.05 (1.9 – 2.2), 11.5
Acetone-d_6	CD_3COCD_3	21.4	2.88	Ketones	Lipophilic, aprotic	2.05 (1.9 – 2.2)
Acetonitrile-d_3	CD_3CN	35.9	3.95	Nitrile	Aprotic	1.95 (6.5 – 5.0)
Dichloromethane-d_2	CD_2Cl_2	9.0	1.60	Chloro	Lipophilic, aprotic	5.32 (5.55 – 5.15)
Dimethyl sulfoxide-d_6	$(CD_3)_2SO$	46.7	3.96	Sulfoxides	Aprotic	2.5
Methanol-d_4	CD_3OD	32.7	1.71	Alcohols	Protic	3.3
Pyridine-d_5	C_5D_5N	13.2	2.21	Amines, aromatcs	Aprotic	7.19,7.55,8.71 (9 – 7)
Deuterium oxide	D_2O	78.3	1.77	-	Protic	4.8
Trifluoroacetic acid-d	CF_3CO_2D	-	1.28	Fluoro	Protic, reactive	11.5

Table S5. Integrals of ^{1}H NMR spectra of dried dissolved organic matter from 9C solubilized in different solvents (% of total integrals).

Core CH substructures (chemical shift, ppm)	Number of scans	Solubility	^{a}DOM-H (0 – 10)	$C_{ar}H$ (7.0 – 10)	O_2CH, CH=CH (5.0 - 7.0)	OCH (3.1 - 5.0)	OCCH, NCH (1.9 - 3.1)	CCH (0.5 - 1.9)
Acetic acid-d_4	12288	Partially soluble	22.1	4.4	6.8	24.2	19.6	45.0
Acetone-d_6	1024	Partially soluble	6.7	15.2	7.5	3.2	6.3	67.8
Acetonitrile-d_3	16384	Partially soluble	5.3	4.4	9.6	9.0	17.8	59.2
Dichloromethane-d_2	12288	Partially soluble	18.5	2.0	1.8	3.5	5.9	86.8
Pyridine-d_5	12288	Partially soluble	3.7	13.1	1.5	20.5	10.7	54.2
Trifluoroacetic acid-d	12288	Reactive/ Fully soluble	-	11.9	13.2	30.9	20.8	23.2

a) The ratio of ^{1}H NMR resonances of the dissolved organic matter and the ^{1}H impurities of deuterated solvent. The ratio for methanol-d_4 and DMSO-d_6 are 67.3 and 22.4 % of total integrals, respectively.

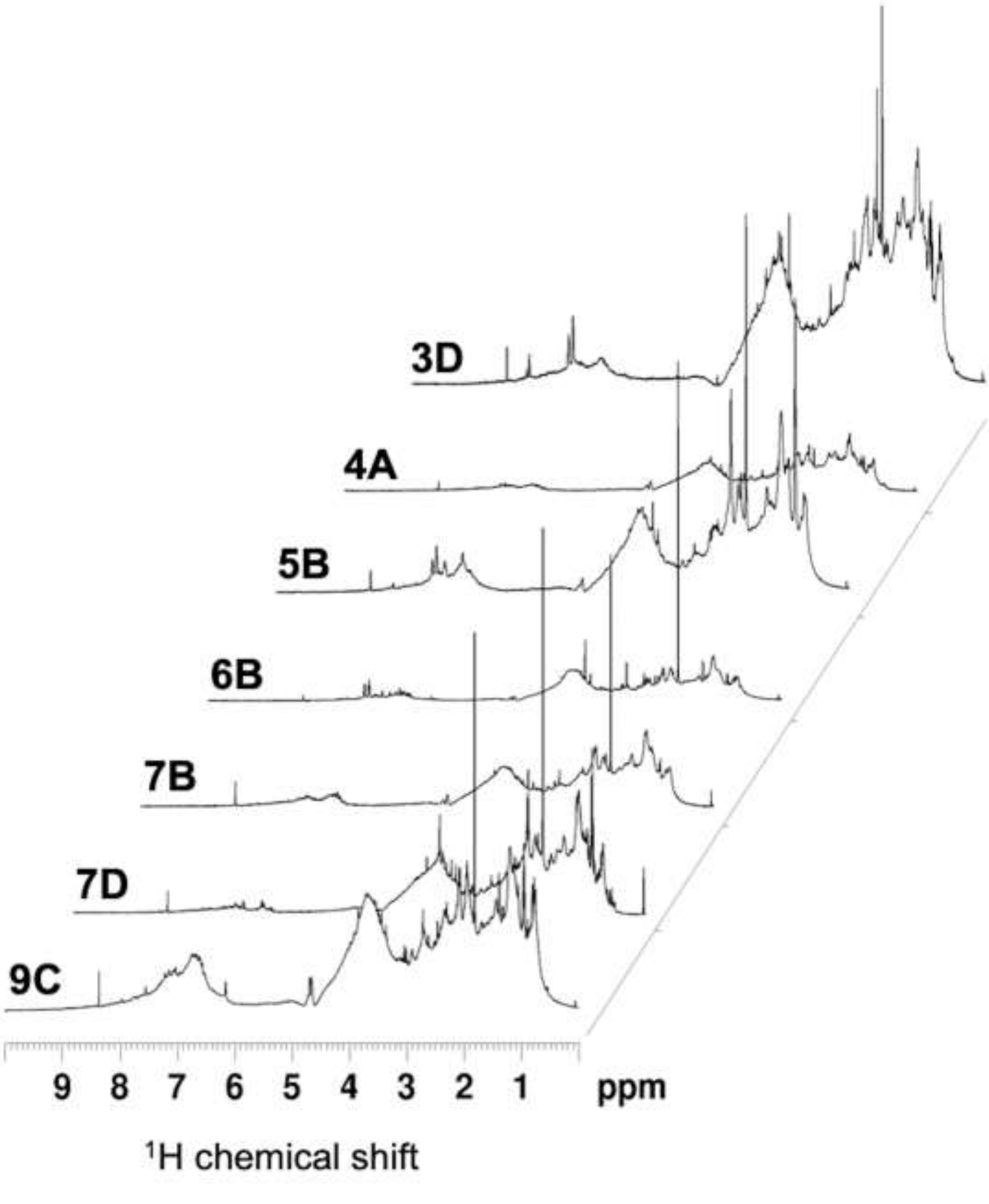

Figure S1. ^{1}H NMR spectra of DOM from seven anaerobic bioreactors in D_2O.

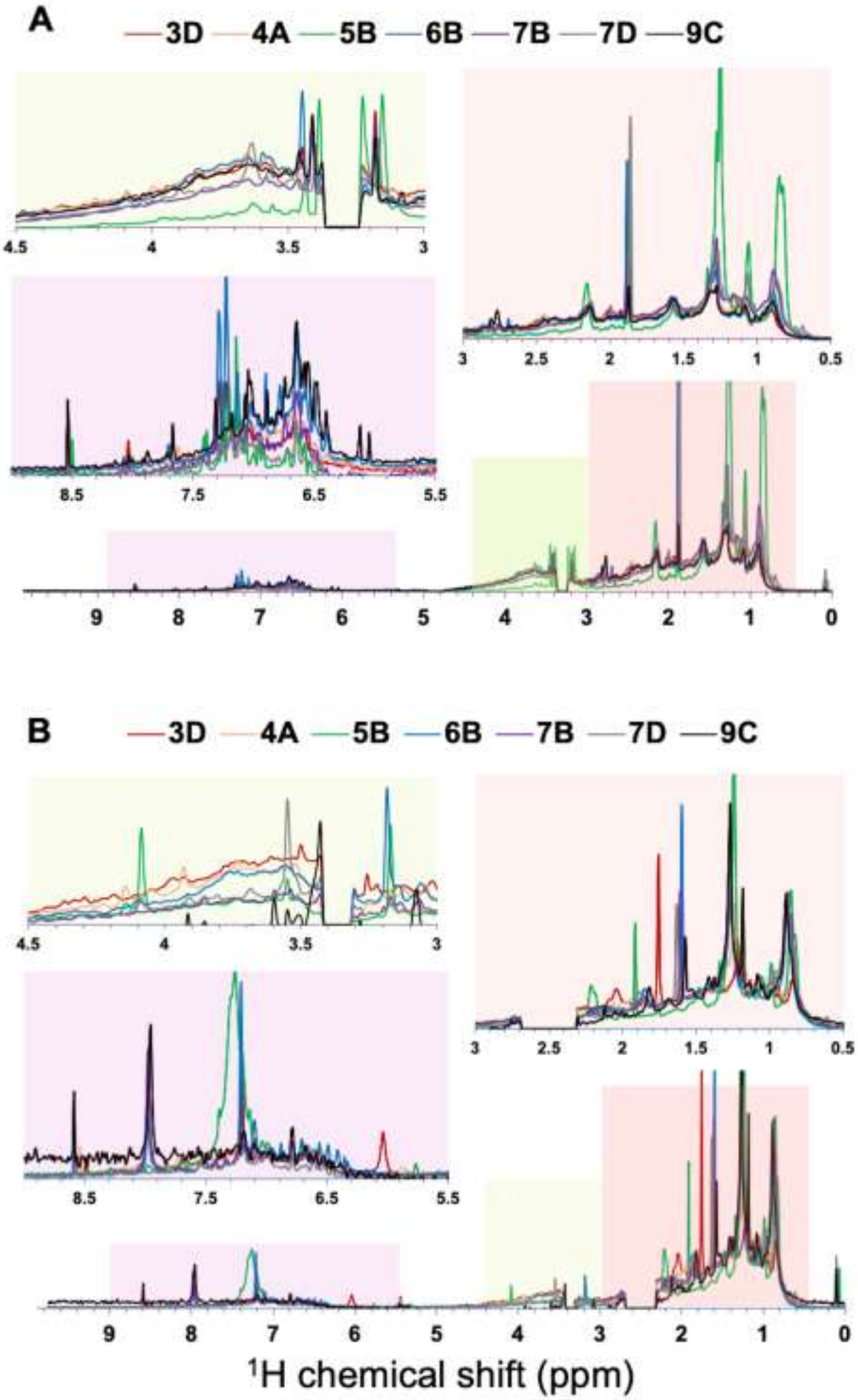

Figure S2. ^{1}H NMR spectra of CD_3OD (A) and DMSO-d_6 (B) extracts from seven anaerobic bioreactors. The solvent signals are removed.

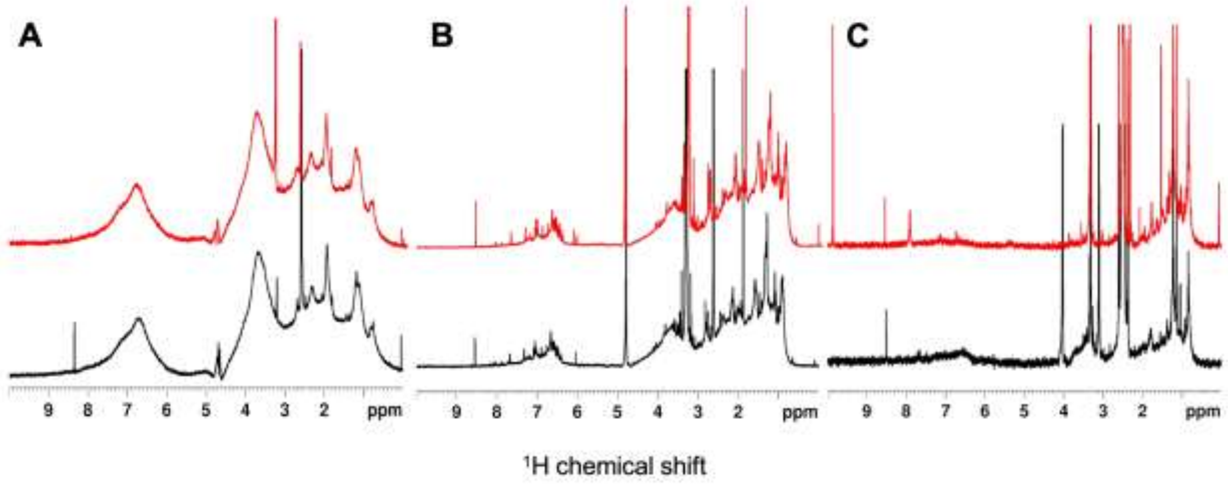

Figure S3. Comparison of ^{1}H NMR spectra of dissolved organic matter from 9C. A) in D_2O after sequential extraction as DMSO-d_6→CD_3OD (red) and CD_3OD→DMSO-d_6 (black), B) in CD_3OD (red) and after sequential extraction as DMSO-d_6→CD_3OD (black), and C) in DMSO-d_6 (red) and after sequential extraction as CD_3OD→DMSO-d_6 (black).

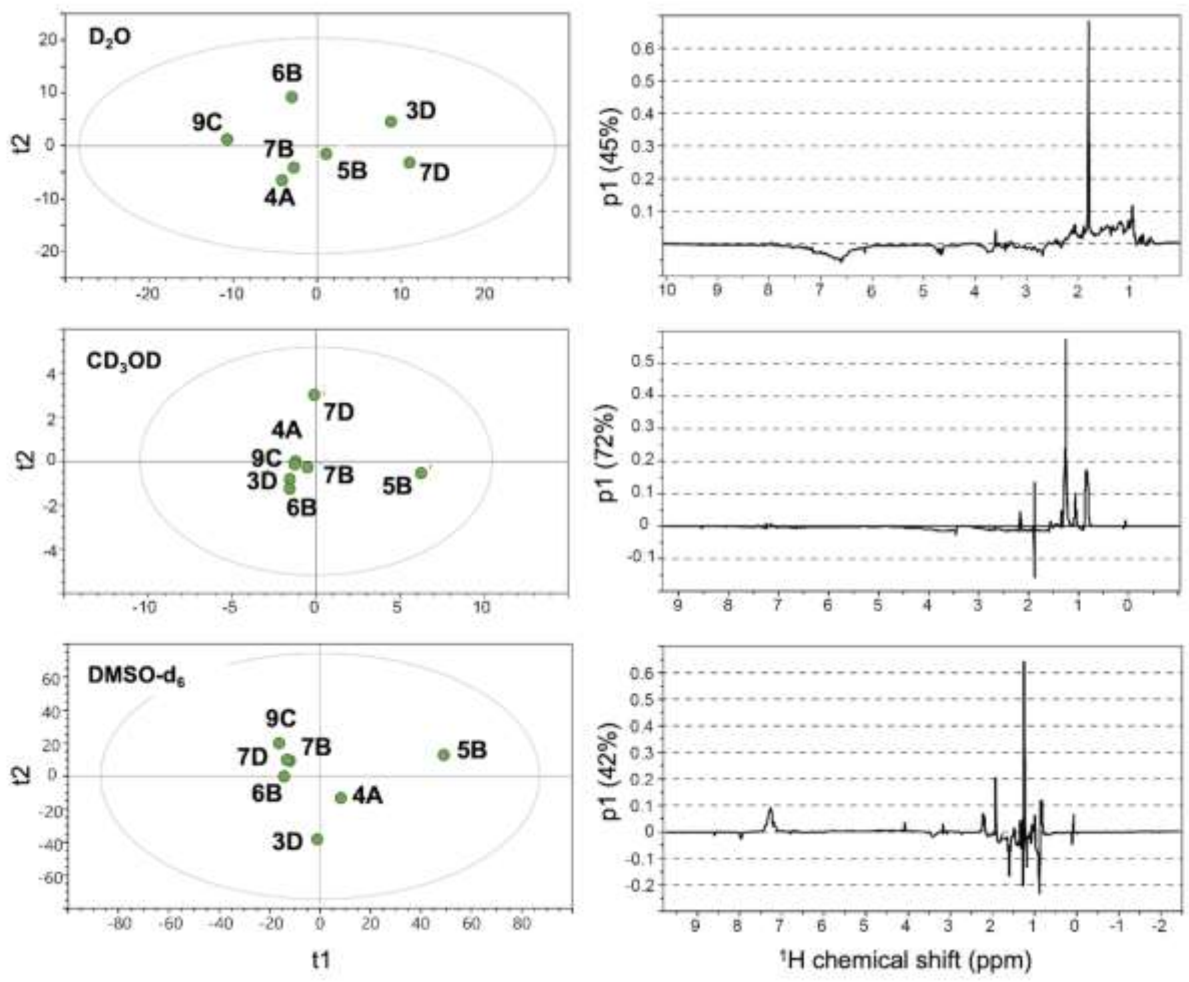

Figure S4. Principal component analyses and first loading plots, p1 (% spectral variance), of the dried dissolved organic matter from seven anaerobic bioreactors solubilized in D_2O, CD_3OD, and DMSO-d_6.

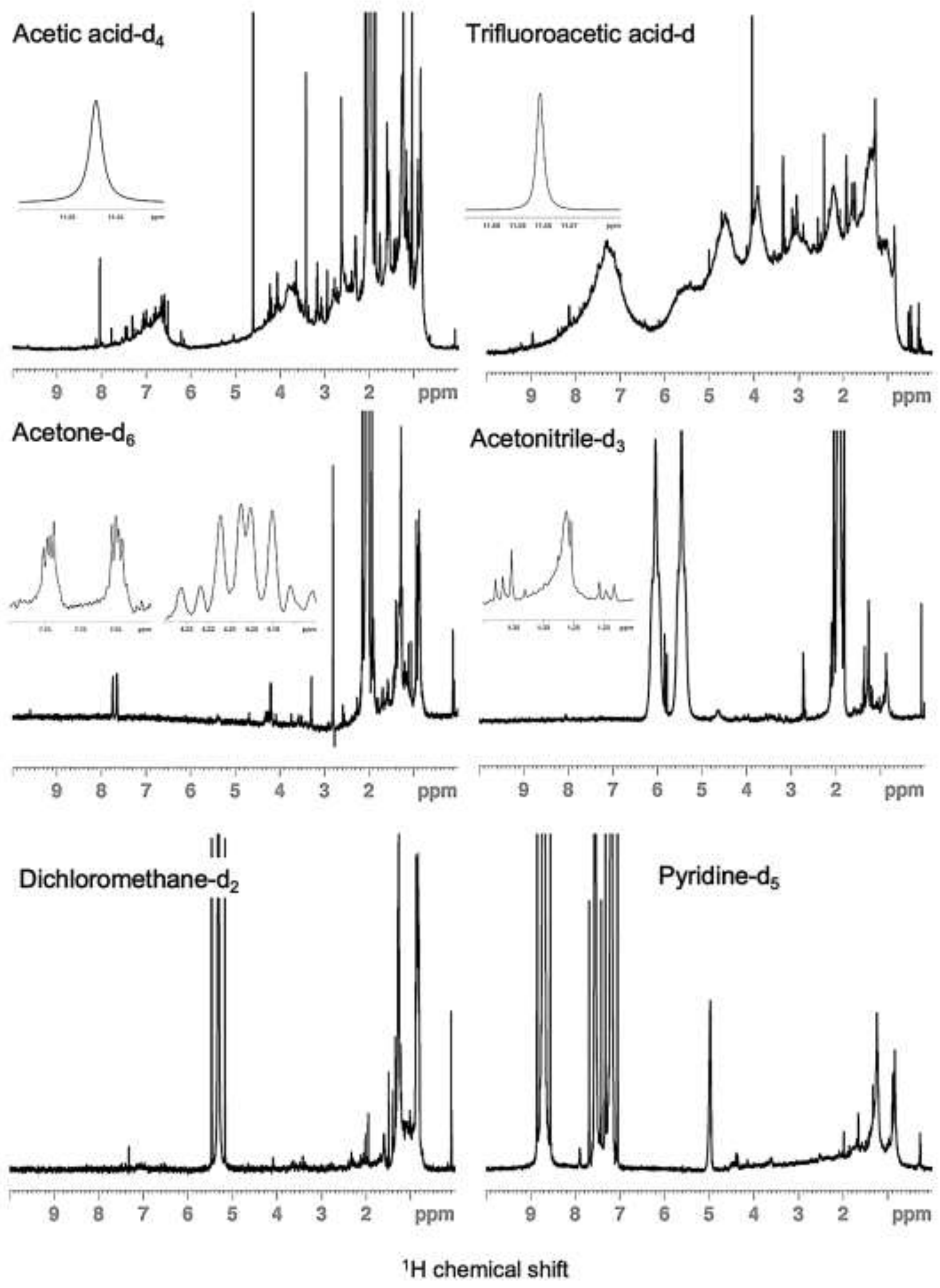

Figure S5. ^{1}H NMR spectra of dried dissolved organic matter from 9C solubilized in different solvents.

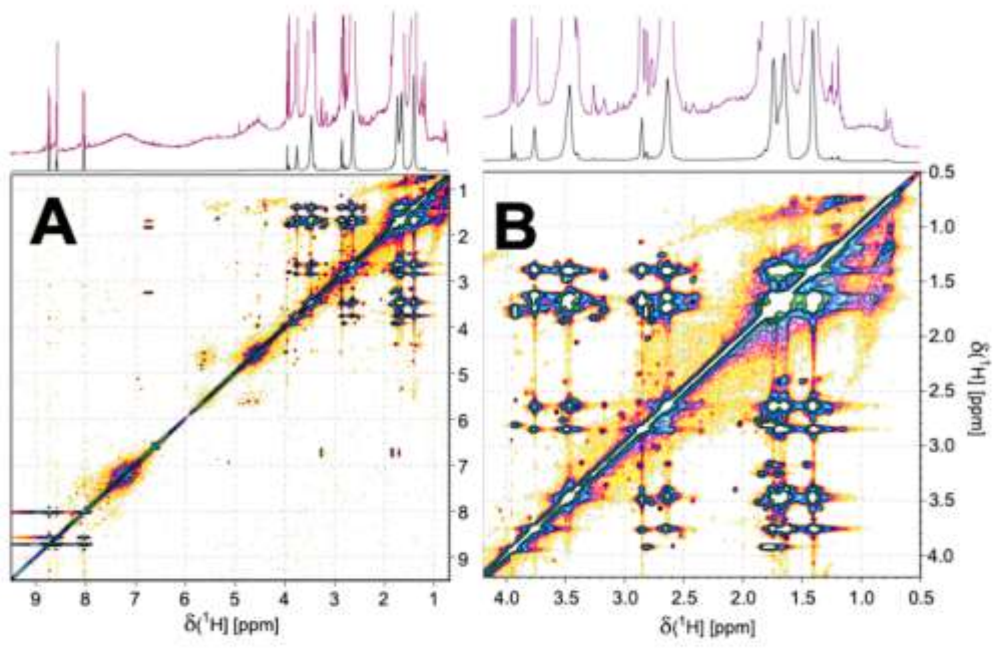

Figure S6. ^{1}H, ^{1}H TOCSY NMR spectrum (850 MHz) of 9C in triflouroacetic acid, CF_3CO_2D, showing strong cross peaks indicative of sample decomposition in the aliphatic region (δ_H ~ 1.2-3.8 ppm). Two major groups of CCC**H** units resonate at δ_H ~ 1.2-1.8 ppm, showing strong cross peaks to OCC**H** units (δ_H ~ 2.5-3.0 ppm), and OC**H** units (δ_H ~ 3.2-3.8 ppm). Termination by methyl is concentrated by cross peaks connecting δ_H ~ 1.2 and 0.8 ppm, indicative of CCC**H**$_3$ units. Strong intra cross peaks indicate numerous H_3C-CH-CH-CH-O- with different remote substitution, which is masked in the one-dimensional ^{1}H NMR spectra, where only a few major broadened resonances dominate. Less abundant but diverse -O-C**H**-C**H**-O- (δ_H ~ 4.2-4.8 ppm), O_2C**H**-C**H**-O- (relating δ_H ~ 4.6 and 5.6 ppm) and -O-C$_{ar}$**H**-C$_{ar}$**H**-C- (δ_H ~ 6.9-7.6 ppm) cross peak contribute a few percent to ^{1}H NMR spectra. Analogous background cross peak integral was also observed in the entire aliphatic region, representing diverse chemical environments. It appears that CF_3CO_2D breaks organic matter into certain major C_{4-6} species, probably directed by positioning of heteroatoms and branching points.

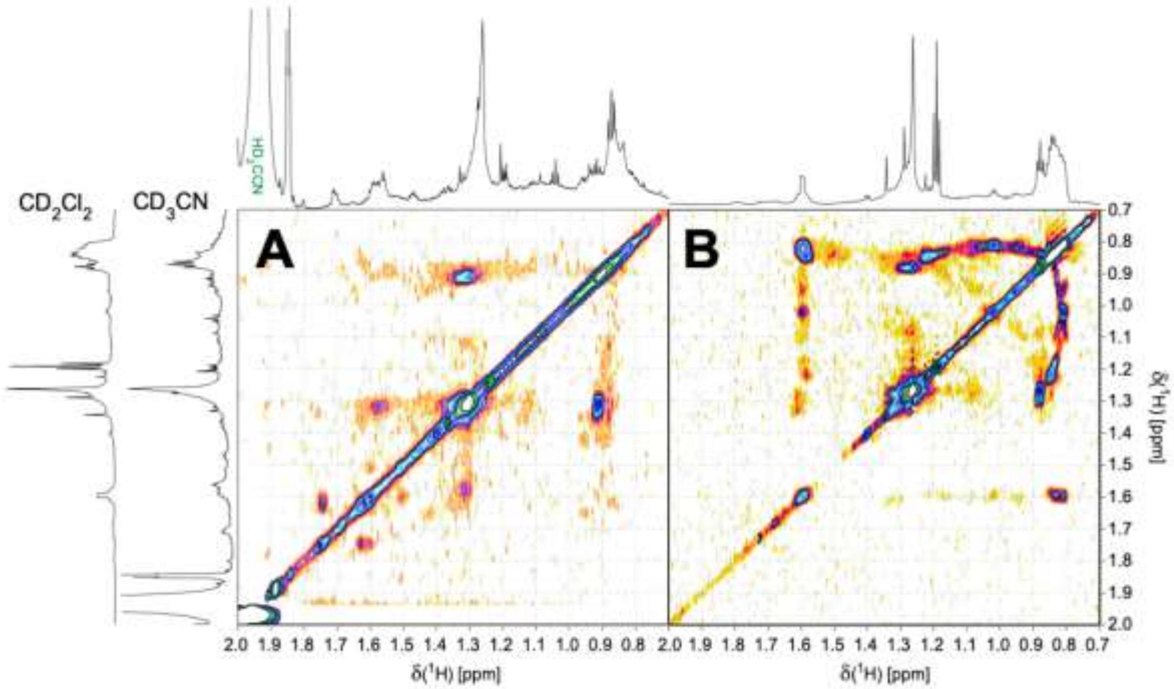

Figure S7. ^1H, ^1H TOCSY NMR spectra (850 MHz) of 9C in (A) acetonitrile, CD_3CN, and (B) dichloromethane, CD_2Cl_2, showing the alkyl region (CCC**H** units). 9C in CD_3CN shows a few well resolved ethyl resonances, with visible J-couplings, and dominant cross peaks indicative of C_3-units (**H**$_3$C-C**H**$_n$-C**H**$_n$; δ_H: 0.92, 1.32, 1.56 ppm). Methyl groups comprise terminal alkyl ($\delta_H <$ 0.95 ppm), but also intra-chain methyl, with methine in α-position to isopropyl derivatives (-C-**H**C(CH_3)-C- units) that resonate at δ_H ~1.1 ppm as well. Intra alkyl cross peaks from branched alkyls resonate from (δ_H ~ 1.3-1.8 ppm) and complement the downfield shoulder at (δ_H ~1.3 ppm) which represents various alkyl units. Alkyls of 9C in CD_2Cl_2 solution are markedly distinct from those in CD_3OD solution, with a distinct ethyl resonance (δ_H: ~0.88, 1.18 ppm), and a broad diversity of methyl resonances at (δ_H ~ 0.8-0.9 ppm), which produce cross peaks to a considerable array of resonances between δ_H ~ 0.9-1.3 ppm, i.e. reflect a larger diversity of branched terminal alkyls than observed in CD_3CN solution. The pronounced cross peak connecting δ_H: 0.84 and 1.6

ppm was not observed in CD_3CN solution, and probably indicates abundant isopropyl and butyl groups, while the cross peak connecting δ_H: 1.2 and 1.6 ppm probably represents -C-(C**H**$_3$)$_2$-C-alkyl units. Overall, 9C in CD_3CN appears to show higher proportions of C_{3-4} linear alkyls, whereas 9C in CD_2Cl_2 shows higher extent of deep alkyl branching, reflected by a higher diversity of terminal methyl chemical environments, including a larger variety of intra chain methyl groups (-C-**H**C(CH$_3$)-C- units) than found in CD_3CN solution.
